# Enhancing residents’ neonatal resuscitation competency through unannounced simulation-based training

**DOI:** 10.3402/meo.v18i0.18726

**Published:** 2013-03-21

**Authors:** Jeffrey W. Surcouf, Sheila W. Chauvin, Jenelle Ferry, Tong Yang, Brian Barkemeyer

**Affiliations:** 1 Division of Neonatology, Department of Pediatrics, Louisiana State University Health Sciences Center–New Orleans, New Orleans, LA; 2 Office of Medical Education and Research and Development, Louisiana State University Health Sciences Center–New Orleans, New Orleans, LA

**Keywords:** simulation-based training, neonatal resuscitation, competency, pediatric residents

## Abstract

**Background:**

Almost half of pediatric third-year residents surveyed in 2000 had never led a resuscitation event. With increasing restrictions on residency work hours and a decline in patient volume in some hospitals, there is potential for fewer opportunities.

**Purpose:**

Our primary purpose was to test the hypothesis that an unannounced mock resuscitation in a high-fidelity in-situ simulation training program would improve both residents’ self-confidence and observed performance of adopted best practices in neonatal resuscitation.

**Methods:**

Each pediatric and medicine–pediatric resident in one pediatric residency program responded to an unannounced scenario that required resuscitation of the high fidelity infant simulator. Structured debriefing followed in the same setting, and a second cycle of scenario response and debriefing occurred before ending the 1-hour training experience. Measures included pre- and post-program confidence questionnaires and trained observer assessments of live and videotaped performances.

**Results:**

Statistically significant pre–post gains for self-confidence were observed for 8 of the 14 NRP critical behaviors (p=0.00–0.03) reflecting knowledge, technical, and non-technical (teamwork) skills. The pre–post gain in overall confidence score was statistically significant (p=0.00). With a maximum possible assessment score of 41, the average pre–post gain was 8.28 and statistically significant (p<0.001). Results of the video-based assessments revealed statistically significant performance gains (p<0.0001). Correlation between live and video-based assessments were strong for pre–post training scenario performances (pre: r=0.64, p<0.0001; post: r=0.75, p<0.0001).

**Conclusions:**

Results revealed high receptivity to in-situ, simulation-based training and significant positive gains in confidence and observed competency-related abilities. Results support the potential for other applications in residency and continuing education.

## Introduction

Four million babies are born in the United States per year. Approximately 10% of all neonates require some level of resuscitation by health care providers at the time of birth, and less than 1% will require advanced resuscitative measures (1, 2). The first course to teach a standardized neonatal resuscitation program (NRP) was developed in 1985 jointly via the American Academy of Pediatrics (AAP) and the American Heart Association (AHA). More than 1.5 million health care professionals have been trained in NRP with regular updates to the program (2). Despite formal NRP education and training, real-life situations often prove difficult to manage. Studies have shown that retention of skills learned in these courses is only 6–12 months (3).

Becoming proficient in the NRP usually requires routine participation in real-life events. The Accreditation Council for Graduate Medical Education (ACGME) and the Residency Review Committee (RRC) allow a maximum of 6 months of intensive care in a pediatric training program (4). Based on ACGME and RRC guidelines, residents can complete pediatric training with only three, and no more than four, rotations in a neonatal intensive care unit, with exposure to patients of variable acuity and inconsistent delivery room experiences (5). Given that new restrictions (e.g., duty hours) in training guidelines have shifted through recent years, residents are finishing training with fewer hands-on experiences in neonatal resuscitation and cite resuscitation management as a deficiency (6, 7). Further, with the increased presence of neonatal nurse practitioners and other health professionals and a decline in patient volume in some hospitals, residents are faced with limited opportunities to gain resuscitation experience (8–10). A survey of pediatric residents in 2000 found that 44% of third-year residents had never led a resuscitation event (6).

In the last decade, simulation-based training has been increasingly adopted for clinical training across the medical education continuum. Such training often immerses trainees in realistic scenarios. These scenarios replicate real-life situations with sufficient fidelity to achieve ‘suspended disbelief’ (11). Such training was first adopted for medical training in the anesthesia community with the use of mannequins for basic life Support training (12–14). Successful use of simulation-based training has been reported more recently in other specialties (e.g., emergency medicine, surgery, obstetrics and gynecology, pediatrics) (15–26). The potential for medical simulation in pediatric residency training is clear, particularly in light of increased national concerns for providing safe, high-quality, affordable patient care, and the declining opportunities for real-life neonatal resuscitation experiences (24, 27, 28). Simulated neonatal resuscitation offers hands-on, guided experience with realistic scenarios that use after-action debriefing that includes both focused and deliberate feedback and practice (10, 26, 29). Consequently, simulation-based training may offer greater opportunity than real-life situations for explicit teaching and learning and the purposeful refinement of relevant knowledge and skills. Further, such training has the potential to improve clinical decision making, sharpen skills, reduce medical error, increase confidence as team leaders, and improve overall teamwork. Conducting such training in the real-life clinical setting also offers the opportunity to identify setting-specific latent conditions (e.g., location of specific resources), practice with real-life team members, and enhance feasibility of embedding such training into the already-packed curriculum and work expectations of busy residents and faculty. The following research questions guided the study:

1. How feasible is it to engage all pediatric and medicine–pediatric residents at all training levels in a NRP in-situ simulation-based training program using unannounced mock codes and a high-fidelity infant simulator (HFIS), the Laerdal SimNewB™?
2. To what extent do residents’ self-confidence for performing critical NRP behaviors change from pre- to post-simulation training assessments, as measured by residents’ responses on pre- and post-training questionnaires?
3. How do residents’ performances change from pre- to post-simulation training, as measured by direct observation conducted by trained assessors using an assessment instrument for critical NRP performance behaviors?
4. To what extent do direct observation-based assessment data correlate with residents’ self-confidence to perform critical NRP behaviors?
5. To what extent do data from video-based assessments correlate with those from direct observation-based assessments of residents’ live NRP performances?

## Methods

Prior to initiation of the program, approval was obtained by the LSU Health-New Orleans Institutional Review Board. The sample was comprised of all 2009–10 Pediatric and Medicine–Pediatric residents at LSUHNO who rotated through Children's Hospital of New Orleans, Louisiana (CHNOLA) during the study period (January–June 2010). Subjects were included if they participated in the unannounced HFIS resuscitation training and voluntarily completed the study questionnaires. Residents were informed about the project prior to initiation, that each would participate in only one session, but they would not know when they would be called to a training session; thus, creating the unannounced mock code feature.

### Training intervention methods

An area of the neonatal intensive care unit (NICU) was set up to support the training environment. Upon arrival to an unannounced mock code, the resident encountered and responded to HFIS resuscitation scenario that required him/her to perform both bag and mask positive pressure ventilation (PPV) and chest compressions in an infant delivered by emergent cesarean section for non-reassuring fetal heart tones. A staff nurse participated as an actor in the scenario to reflect a real-life situation. Resuscitation proceeded up to 10 min followed immediately by a structured debriefing session in the same setting between the resident and faculty trainer, lasting 10–15 min (30). The scenario–debriefing cycle was repeated with a second scenario that required the resident to perform neonatal resuscitation involving PPV and intubation due to respiratory distress associated with meconium-stained amniotic fluid. Following the debriefing of the second simulation scenario, the training session concluded with an emphasis of key targets for enhancing future performance. The total length of a training session was one hour.

In the focused debriefings, the faculty trainer/facilitator prompted the resident to reflect on what went well and what could be improved. The trainer also provided specific feedback and coaching, targeting specific improvement strategies to focus practice on enhancing targeted knowledge and behaviors/skills. Closure was achieved by summarizing the scenario-based experiences, reinforcing strengths, and targeting specific strategies for further improvement. Because debriefing occurred in the simulation setting, video recordings were not necessary to achieve accurate recall. Residents were asked to honor the confidentiality of the session and scenarios by not discussing these with anyone outside of the session.

### Training evaluation methods

Kirkpatrick's four levels of training effectiveness provided a model for program evaluation (31). Residents completed pre- and post-training questionnaires (Level 1: reaction, motivation; Level 2a: change in confidence/perceived competence). Direct observations of residents’ performance of critical NRP behaviors was conducted by trained assessors during training, and later, by assessment of video-recorded performances conducted by a single content expert (i.e., ‘Gold Standard’) who was a content expert for instrument development (Level 2b: change in knowledge; Level 3: behavior change). For logistical reasons, we were not able to assess Level 4 outcomes (real-life impact, outcomes).

### Instrumentation

The pre- and post-training questionnaire contained several demographic items (e.g., prior experience in NRP, PGY level) and 14 specific behaviors reflecting adopted NRP best practices derived from literature review and input from content experts experienced with resident education (e.g., stimulating and drying, administration of supplemental oxygen, positive pressure ventilation, intubation, and chest compressions). Residents responded to the 14 NRP items using a four-point Likert-type scale (0=not confident at all to 4=completely confident) that yielded a maximum possible total score of 56. Residents responded to the same 14 NRP items on the post-training questionnaire and also to 10 items pertaining to session effectiveness (5-point, Likert-type scale, 1=Definitely no to 5=Definitely yes). Three open-ended questions provided additional opportunities for residents to comment on their desire for future training, input on increasing realism of scenarios, and other feedback for improving training sessions. The direct observation assessment instrument used for both live and video-recorded resident performances included the specific behaviors reflected in 14 NRP critical behaviors and additional performance quality and time measurements defined for specific NRP behaviors, yielding 23 discrete assessment judgments. The overall structure of the instrument was an adaptation of an assessment protocol developed and used previously for evaluating simulation-based training in pediatric resuscitation using unannounced mock codes in which the instrument demonstrated good psychometric qualities. Scoring of the observation-based assessments yielded a maximum possible total score of 41.

### Data collection

Residents completed the pre-program questionnaire at the start of the project period before any training was initiated. Residents completed the post-training session questionnaire immediately upon completion of their respective training event. For each questionnaire, residents followed directions to generate a record identification code for matching individual responses across measures, while maintaining anonymity. At each training session, one trained observer only recorded the directly observed behaviors and did not assign any numerical scores. A senior member of the team representing an expert clinician (‘gold standard’) completed a separate and independent observation-based assessment of each video-recorded resident performance using the same instrument that was used in the live session. All observation-based assessments were subsequently scored by two members of the research team using a standard ‘grading’ rubric reflecting the critical NRP behavior standards. Three trained assessors were neonatology Fellows, of which one was a senior neonatology faculty member who served as the ‘Gold standard’ content expert.

### Data analysis

After data compilation and initial screening, descriptive statistics were calculated. Data for the pre- and post-self-confidence questionnaire responses were examined using the Wilcoxon Signed Rank Test. Content analysis was used to examine narrative responses to open-ended questions for common perspectives. Comparison of residents’ performances in scenarios 1 and 2 was accomplished using the paired *t-*test for observation-based assessment data. To examine relationships between residents’ self-confidence and actual observed performance and between observation data for live and video-based assessments, correlational analyses were conducted.

## Results

Results are reported by the research questions set forth at the beginning of this article.

### Question 1: Feasibility of training

Each of the 32 residents rotating through CHNOLA during the study period completed one training session and participated voluntarily in the study (100%, 11 PGY-1, 11 PGY-2, 10 PGY-3). One faculty member was able to conduct one or more *in situ* unannounced mock code training sessions per training day. No narrative feedback from residents or feedback from faculty and staff participants indicated any significant interruption or inconvenience with other typical responsibilities.

### Question 2: Change in residents’ self-confidence

**Fig. 1:**
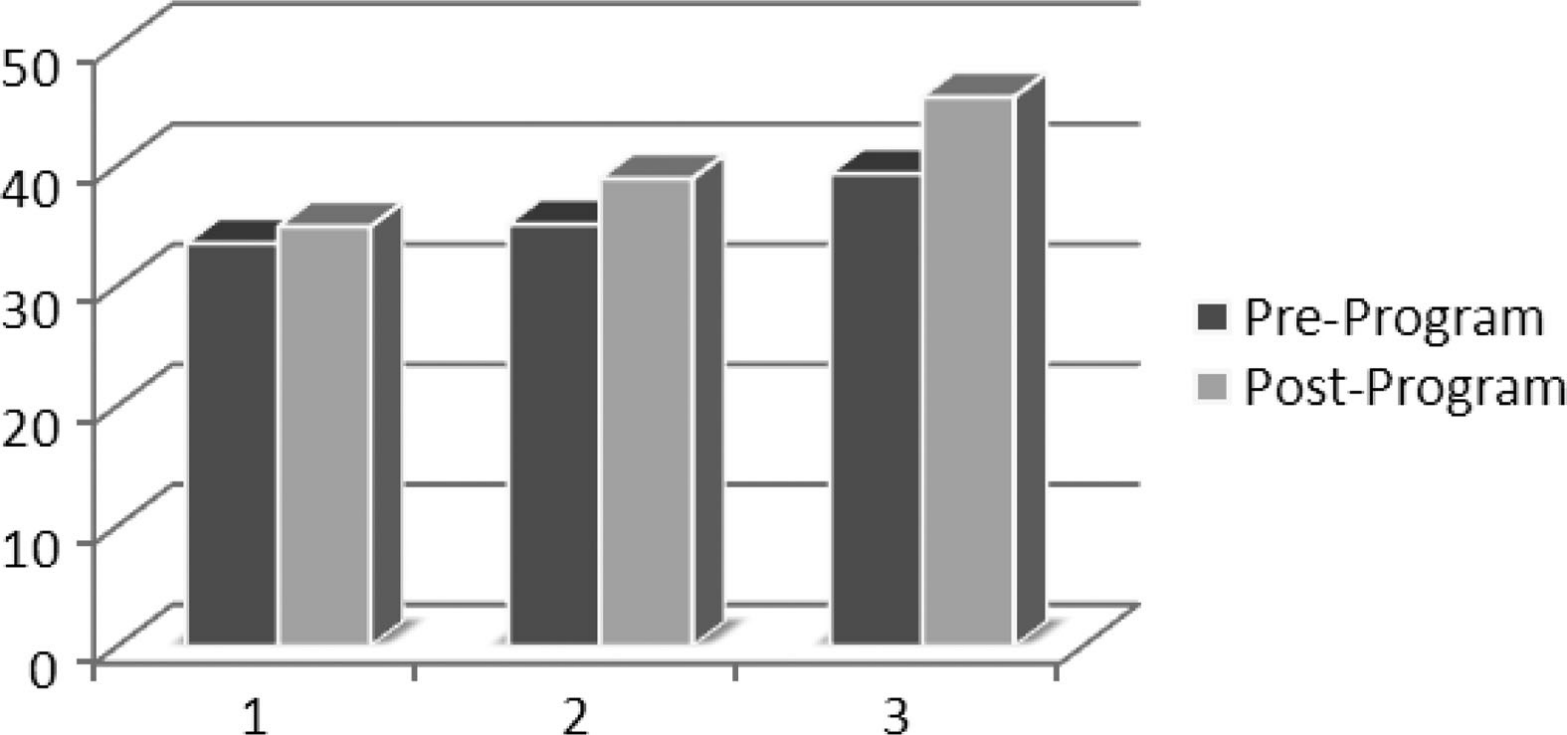
Difference in self-assessments per postgraduate year of training. The x-axis represents the year of training and the y-axis represents the mean total score for pre- and post-program self-reported self-confidence.

Pre-training data were not available for five residents due to a logistical glitch, so the analysis included matched pre/post data for 27 residents. Table 1 shows positive gains for 13 of the 14 self-confidence items, with statistical significance for 8 (Wilcoxon signed rank test, p=0.00–0.03) and for the total scale score (p=0.00): Analysis of performance by post-graduate year in training revealed positive pre–post gains for all three groups, but results were not statistically significant (Fig. 1).

**Table 1:** Results for residents’ responses on the pre- and post-NRP simulation training questionnaire

	Pre	Post	Change†	p‡
Explain NRP algorithm	2.29 (0.71)*	2.66 (0.66)	0.37	0.030
Perform as an effective team member	2.89(0.50)	3.56 (0.62)	0.67	0.000
Perform as effective team leader	2.00 (0.72)	2.93 (0.78)	0.93	0.000
Communicate effectively	3.00 (0.67)	3.44 (0.56)	0.44	0.010
Stimulate and dry	3.71 (0.46)	3.86 (0.50)	0.15	0.346
Administer bag-mask ventilation	3.39 (0.63)	3.69 (0.54)	0.30	0.080
Place endotracheal tube	2.29 (0.71)	3.03 (0.71)	0.74	0.000
Perform chest compressions	3.04 (0.58)	3.45 (0.72)	0.41	0.003
Request appropriate drugs	2.14 (0.76)	2.58 (0.96)	0.44	0.005
Administer correct dosages	1.93 (0.77)	2.23 (0.99)	0.30	0.137
Use appropriate methods to administer drugs	2.18 (0.77)	2.55 (0.96)	0.37	0.020
Decide when to place umbilical venous line	1.89 (0.83)	2.11 (0.88)	0.22	0.186
Execute correct placement of umbilical line	1.54 (0.79)	1.87 (0.85)	0.33	0.057
Assign APGAR score	3.11 (0.69)	2.92 (0.65)	−0.19	−0.227
Overall mean	2.53 (0.46)	2.92 (0.56)	0.39	0.000

* Mean (Standard Deviation).

† Change=post-minus pre-training score.

‡ Statistical significant set at p≤0.05.

### Question 3: Change in residents NRP performance, based on direct observation assessment

**Fig. 2:**
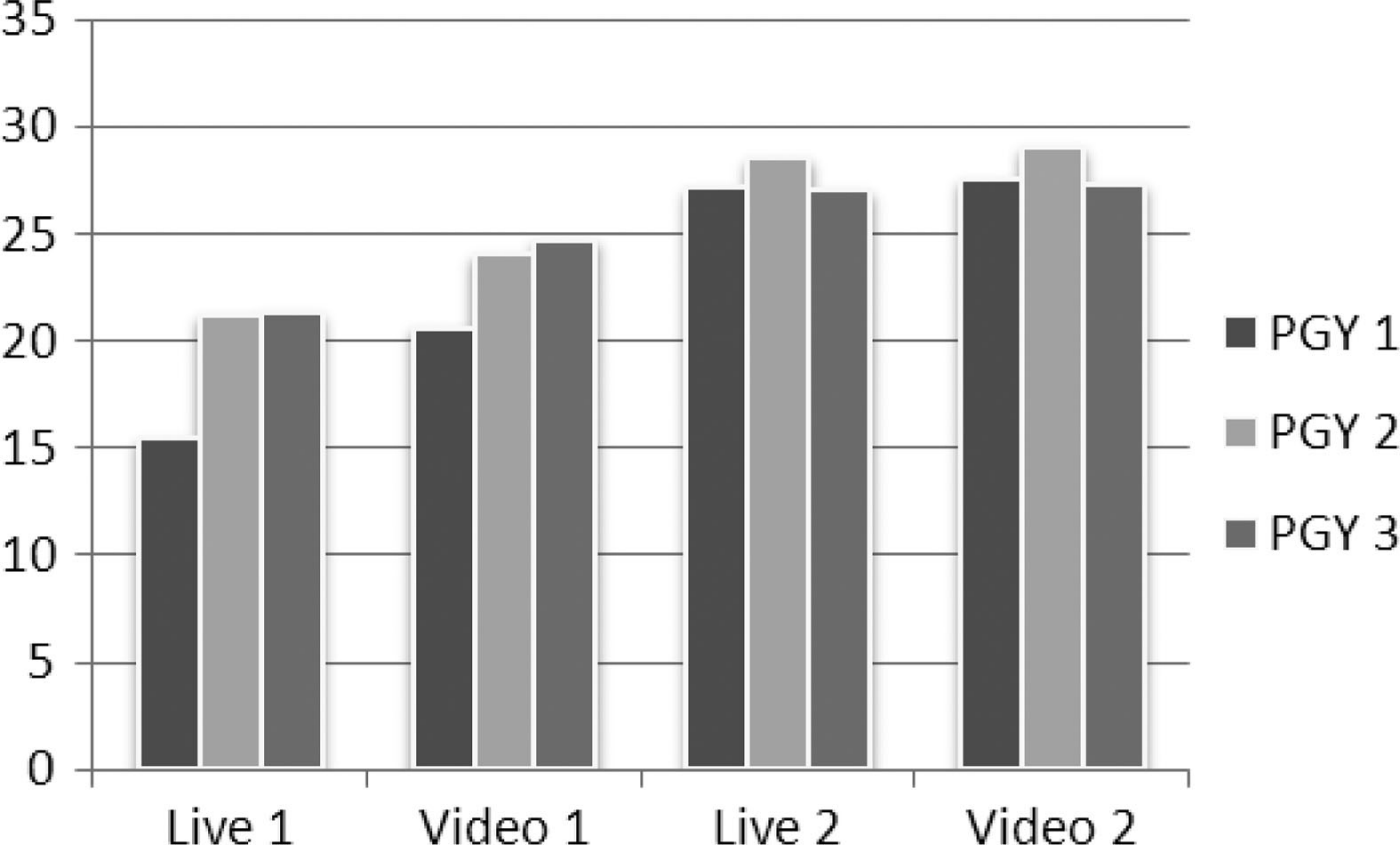
Difference in observed performance by post-graduate year of training for live and video-based assessments. The x-axis represents performance by year of training for live and video-based assessments. The y-axis represents the mean total score for observed critical performance indicators for scenarios 1 and 2.

With a maximum possible total score of 41, the average pre–post gain was 8.28 and statistically significant (paired *t*-test, n=32, p<0.0001). Results of the observation-based assessments using a video recording also revealed statistically significant performance gains (p<0.0001). Results showed an improvement in response time and quality of actions from the first simulation scenario to the second. The greatest improvement was found in first-year trainees (PGY-1). The average performance scores for each of the two scenarios, represented per year of training, are shown in Fig. 2.

### Question 3: Correlation between self-reported self-confidence and observed performance

The correlation between self-confidence and actual performance was not statistically significant (pre: r=0.12, p<0.55; post: r=0.06, p<0.76).

### Question 4: Correlation between live and video-based observation assessments

Correlation between the live and video-based observations was strong for both pre- and post-training scenario performances (pre: r=0.64, p<0.0001; post: r=0.75, p<0.0001). The differences in total scale scores between live and video-based observations were greater for pre than post-training performances (pre: 3.82, p=0.0011; post: 0.43, p=0.43). Pre- to post-training gains were noted for both live and video-based observations, and item ratings were generally higher for video-based than live assessments.

## Discussion

Several limitations of this study are worthy of mention. First, the study occurred in one setting at one institution with pediatric residents. Second, the study did not include a control group, but used residents as their own control through pre–post training measures. Given the existing structure of residents’ educational program and limited hands-on NRP experience, and faculty agreement that NRP knowledge and skill was limited, priority was given to providing training to all currently enrolled residents. Third, training evaluation included measures addressing Kirkpatrick's Levels 1–3 (i.e., reaction, self-confidence, performance in simulated setting), but Level 4 measurement of actual practice outcomes in the real-life setting was not possible because of logistical constraints. The extent to which the results generalize to other pediatric residency programs will depend on the similarity of contexts and residents’ prior experiences in neonatal resuscitation. Also, replication of the curriculum and training with an intervention and control group and the inclusion of Level 4 outcomes measurement would contribute to strengthening the program evaluation design and resulting evidence from conclusions could be generalized to other pediatric residency settings.

Regarding Question 1, results demonstrated that the *in situ* NRP simulation-based training program was logistically feasible. Training fit well within the context of the existing curriculum and clinical care requirements. Using an unannounced/unscheduled format provided the flexibility necessary to complete activities within the context of the residents’ schedule and the requirements of a typical work day within a reasonable study time frame. Most residents responded positively to the unannounced mock NRP simulation training.

Results pertaining to Question 2 (self-reported self-confidence) provided strong evidence of the efficacy of simulation-based training for NRP. While self-confidence is not entirely a proxy for actual performance in real-life settings, its contribution should not be under-estimated. According to Bandura's construct of self-efficacy and the work on developing expertise by Ericsson et al., self-confidence is a significant influence on individuals’ motivation and self-regulation of behavior (29, 32–35). Further, there is increasing evidence that self-confidence is an important mediating factor that contributes to the extent to which one approaches learning and persists toward achievement of goals and expertise (29, 31–38). High fidelity simulation is an expensive technology for instruction and assessment, but a key contribution is its ability to support an authentic environment, particularly *in situ*, for learning and assessing clinical skills when real-life experiences are either insufficient or inappropriate for teaching, learning, and assessment. In the context of developing future physicians, self-confidence is an important measure at certain points within a curriculum and for certain learning experiences (e.g., when and how to incorporate simulation-based training). Consequently, from an educational and practical perspective, the statistically significant gains in self-confidence for 8 of 14 NRP critical performance behaviors are considered an important contribution of this study.

The results pertaining to Question 3 (correlation between self-reported self-confidence and actual observed performance) reiterate the caution previously communicated about using self-confidence as an overall proxy measure for actual performance or behavior change. However, as mentioned in the previous paragraph, there are other valid reasons for attending to and measuring self-confidence in the teaching and learning process. Upon closer examination of the data, we observed that third-year residents’ self-confidence ratings were higher than those of less experienced residents, perhaps simply reflecting a higher level of motivation and persistence to strive toward achievement and expertise, given their closer proximity to graduation and entry into professional practice. Similarly, we observed that three of the first-year residents reported less self-confidence on post-training than pre-training, possibly an indication of their inflated espoused abilities prior to having hands-on experiences in real-life and simulation scenarios. Their post-training responses may be indicative of achieving a more accurate self-appraisal that resulted from the simulation-based training. This may be an important consideration for the use of simulation-based training as a strategy for enhancing both individual motivation and self-regulation in professional learning and for improving safety in patient care for the future.

Question 4 was important to consider the feasibility of using video-based observation as an accurate alternative to conducting assessment during the live simulation-based training sessions. We recognize that seeing and hearing performances is easier and more accurate in the live observations, as the placement and fidelity of video cameras cannot substitute for the incredible abilities of the human senses. The positive and statistically significant correlation between live and video-based assessments was encouraging. The higher correlation between live and video assessments for observations of post-training performances may have been due the residents’ more explicit or enhanced demonstration of specific behaviors in post-training performances or it may have been due to the influence of practice on assessors’ abilities in observing and recording judgments on the assessment instrument. With increased demands and time constraints, the use of a video-based approach for performance-based assessment appears to be a practical option, at least for low-stakes, informal assessments used for coaching and providing formative feedback.

## Conclusions

In summary, we are encouraged by the study results regarding the development of a sustainable, standardized curriculum to enhance NRP competency in pediatric residents. While self-confidence and demonstration of improved abilities in a realistic, simulated scenario is important, there is still the need to include examination of the transfer of learning and abilities to real-life practice and to relevant outcomes (e.g., patient, team, organizational). Furthermore, the NRP simulation-based training model may be a viable option for maintaining adequate levels of competency for specific clinical knowledge, technical, and non-technical skills reflected in low frequency, high stakes situations or when learning and training in procedural skills is not feasible or appropriate in the context of real patient care. In addition to residency training, such use of in-situ and center-based simulation could be highly relevant for physicians in practice, particularly in certain types of communities where frequency and consequence may be even more critical (e.g., rural, critical access hospitals, medically underserved communities) (24–26, 39, 40). Members of the research team have begun to explore this area of simulation-based training. Another area of simulation-based training that still needs further attention is the extent to which gains achieved are maintained and how much time must lapse before knowledge and skills degrade to a point when re-training or re-calibration is necessary to reinforce or sustain competency? What type and to what extent must follow-up training reflect the original format of training? These and other questions are opportunities on which to build upon this work and the work of others in simulation-based training for clinical knowledge and skills development across the medical education continuum.
